# Comprehensive analysis of co-occurring domain sets in yeast proteins

**DOI:** 10.1186/1471-2164-8-161

**Published:** 2007-06-11

**Authors:** Inbar Cohen-Gihon, Ruth Nussinov, Roded Sharan

**Affiliations:** 1 Sackler Institute of Molecular Medicine, Department of Human Genetics, Sackler Faculty of Medicine, Tel Aviv University, Tel Aviv, Israel; 2 Center for Cancer Research Nanobiology Program, SAIC-Frederick, Inc., NCI-Frederick, Frederick, Maryland 21702, USA; 3 School of Computer Science, Tel Aviv University, Tel Aviv 69978, Israel

## Abstract

**Background:**

Protein domains are fundamental evolutionary units of protein architecture, composing proteins in a modular manner. Combinations of two or more, possibly non-adjacent, domains are thought to play specific functional roles within proteins. Indeed, while the number of potential co-occurring domain sets (CDSs) is very large, only a few of these occur in nature. Here we study the principles governing domain content of proteins, using yeast as a model species.

**Results:**

We design a novel representation of proteins and their constituent domains as a protein-domain network. An analysis of this network reveals 99 CDSs that occur in proteins more than expected by chance. The identified CDSs are shown to preferentially include ancient domains that are conserved from bacteria or archaea. Moreover, the protein sets spanned by these combinations were found to be highly functionally coherent, significantly match known protein complexes, and enriched with protein-protein interactions. These observations serve to validate the biological significance of the identified CDSs.

**Conclusion:**

Our work provides a comprehensive list of co-occurring domain sets in yeast, and sheds light on their function and evolution.

## Background

Protein domains are fundamental evolutionary units of protein architecture. They function as independent units and occur in different combinations, formed by duplication, divergence and recombination of genes. In spite of their modularity, the actual number of combinations is only a small fraction of the number of potential combinations, mainly since the evolution of the protein repertoire is based on the expansion of existing protein families rather than on *ab initio *formation of new proteins [1].

While there is no doubt that the functionality of a protein is derived from its domain composition, the laws governing the domain content of proteins are still largely unknown. The recent availability of large-scale data on the domain content of proteins (in the form of sequence signatures [2]) allows us to ask fundamental questions regarding protein architecture: What are the common attributes of proteins sharing certain domains? Are domains used independently, or do they form synergistic combinations?

Studies of the combinatorics of domain organization have shown that there are many kingdom-specific two-domain combinations of common domains and that recombinations of these common domain families have been a key factor in the divergence of organisms [3]. Vogel et al [4] studied combinations of adjacent pairs or triplets of domains, referring to those as *supra-domains*. About half of the supra-domains were found to be overrepresented within proteins in all kingdoms of life; moreover, these combinations occurred within proteins involved in a variety of functions like metabolism, regulation and others. A follow-up study suggested that these combinations are formed once during evolution of the protein repertoire and are duplicated as a single evolutionary unit [5].

Wuchty et al. [6] and Ye et al. [7] studied domain combinations within proteins using a co-occurrence network of domains, where two domains are linked if they are found within the same protein. Wuchty et al. showed that many domain co-occurrence networks have a giant component containing the vast majority of the nodes. A comparison of domain networks across several genomes revealed that there are similar numbers of domains in higher and lower eukaryotes, while the sizes of highly connected domain subgraphs grow with evolution. This suggests that the increasing complexity of multicellular organisms relates to the formation of new domain combinations. Ye et al. partitioned the co-occurrence network of domains into clusters and showed that domains within the same cluster tend to have similar functions.

Betel et al. [8] devised a method to identify pairs of domains from different proteins that tend to co-occur within the same protein complex. They studied the global properties of the resulting domain networks from two different protein complex sources: manually curated and large scale experiments, and found different topologies for these data sources. The former contained large sub-networks corresponding to known biological assemblies, like ribosomal subunits. The latter was typically small-world and contained a few central hubs, mainly of RNA processing and binding domains. Hegyi and Gerstein [9] investigated the functional similarity of proteins that share domains. They found that about 80% of protein pairs sharing the same domain combination also share the same function. They further showed that about two-thirds of single-domain proteins that share the same domain have the same function. On the other hand, they found that only 35% of multi-domain protein pairs that share only a single domain, have the same function. Müller et al [10] suggested that changing the repertoire of domain partners in a combination, along with refinement and diversification of the domain repertoire, increases functional complexity.

Other related works focused on identifying and analyzing domain-domain interactions. Several works aimed at inferring domain interactions from protein interactions [11,12] or integrating domain and protein interactions to better explain interactions at the domain level [13]. Others explored the interactions between families of domains, revealing that interactions within families are significantly more frequent than between families [14], or associated between domain interactions and their co-occurrence within proteins in other organisms [15].

Here we perform a comprehensive study of the domain composition of proteins in yeast. First, we study single domains, characterizing sets of proteins sharing each domain and the distribution of domain connectivities. Second, we use a novel network representation of the domain data to identify combinations of domains that co-occur in proteins more than expected by chance. In difference from previous works, our framework allows the identification of combinations of any size; moreover, these combinations are allowed to occur non-contiguously along the protein. We study the functional significance of these combinations, which we term *co-occurring domain sets (CDSs)*, and the sets of proteins they induce.

## Results

### Bipartite graph representation of proteins and domains

**Figure 1:**
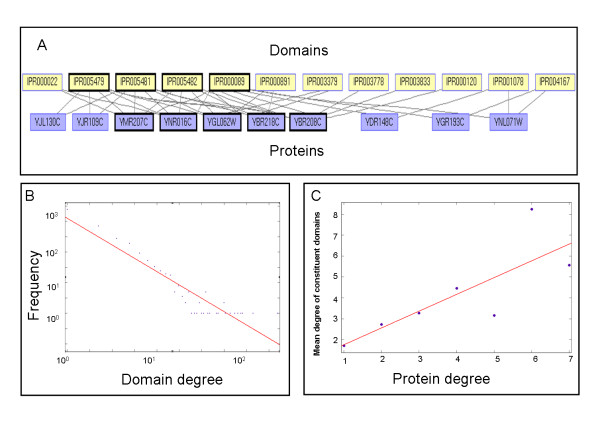
Bipartite graph representation of proteins and domains. **(A) **An example of a subgraph of metabolic enzymes and their constituent domains. Nodes correspond to proteins and domains. Edges connect proteins to their constituent domains. In bold is a biclique with five proteins and four domains. The biclique's proteins include acetyl-CoA carboxylase, pyruvate carboxylase 1 and 2, urea amidolyase and HFA1 (SwissProt IDs YNR016C, YGL062W, YBR218C, YBR208C and YMR207C). The proteins share four domains: Carbamoyl-phosphate synthase L chain, Carbamoyl-phosphate synthetase large chain N-terminal, Biotin carboxylase C-terminal and Biotin/lipoyl attachment (Interpro IDs IPR005479, IPR005481, IPR005482 and IPR000089). **(B) **Distribution of number of proteins per domain in the data. Regression line parameters: f(x) = -1.74x + 2.7 (linear fit model *p < 0.0001*). **(C) **Correlation between domain and protein degrees. For each degree of a protein, ranging from 1 to 7, the mean degree of domains comprising these proteins was calculated. Regression line parameters are f(x) = 0.8x + 0.96 (linear fit model *p < 0.0327*).

We analyzed the domain content of 3,321 *S. cerevisiae *proteins annotated with 1,588 domains from the Interpro database [2]. We represented these data using a bipartite graph, whose nodes correspond to proteins and domains, and whose edges connect proteins to their constituent domains (Figure 1A and Methods). In agreement with previous studies [16,17], we found the distribution of the number of connections (*degree*) per protein to be exponential (data not shown). In contrast, we found that the degree distribution of domains follows a power law (Figure 1B; *p < 0.0001*).

Next, we investigated the relation between protein degree and domain degree. We identified a significant positive correlation between the degree of a protein and the degrees of its constituent domains (Figure 1C): Multi-domain proteins tend to consist of abundant domains, i.e., domains that are found within at least four proteins. On the other hand, single-domain proteins tend to contain rare domains (*p < 0.0238 *by Spearman correlation test). When further comparing the distributions of domain degrees in single-domain and multi-domain (having 3 domains or more) proteins, we observed that the distributions are significantly different (*p < 0.0013 *by a Wilcoxon rank sum test). Interestingly, a recent study [18] reported that hub proteins, having many interacting partners, tend to be multi-domain. In agreement with that study, we found that hub proteins in the yeast PPI network are significantly multi-domain, compared to non-hub proteins (*p < 7.9e-6*, hypergeometric score, see Methods). Furthermore, the average degree of domains within these hubs was significantly larger than that for non-hubs (4.414 vs. 2.68; *p < 8.5e-6 *by a hypergeometric test). In light of this finding, our results suggest that the abundant domains found in multi-domain proteins may be important for their protein-protein interaction ability.

### Co-occurring domain sets

We used the graphic representation to explore the repertoire of CDSs within yeast proteins. In the protein-domain bipartite graph representation, such a combination is represented by a *biclique *(a fully connected bipartite subgraph, see Figure 1A). Specifically, a biclique on a set of proteins *P *and a set of domains *D*, implies that every protein in *P *contains all the domains in *D *and, hence, suggests *D *as a functional CDS. The more proteins in *P *the more support for the combination *D*. We score a biclique according to the chance of observing it at random (see Methods). The problem of searching for the highest-scoring biclique in our setting is computationally hard [19]; nonetheless, we exploit the fact that each protein contains a relatively small number of domains (up to 7 in our data) to derive an efficient algorithm for identifying all the significant bicliques in the network (Methods).

**Figure 2:**
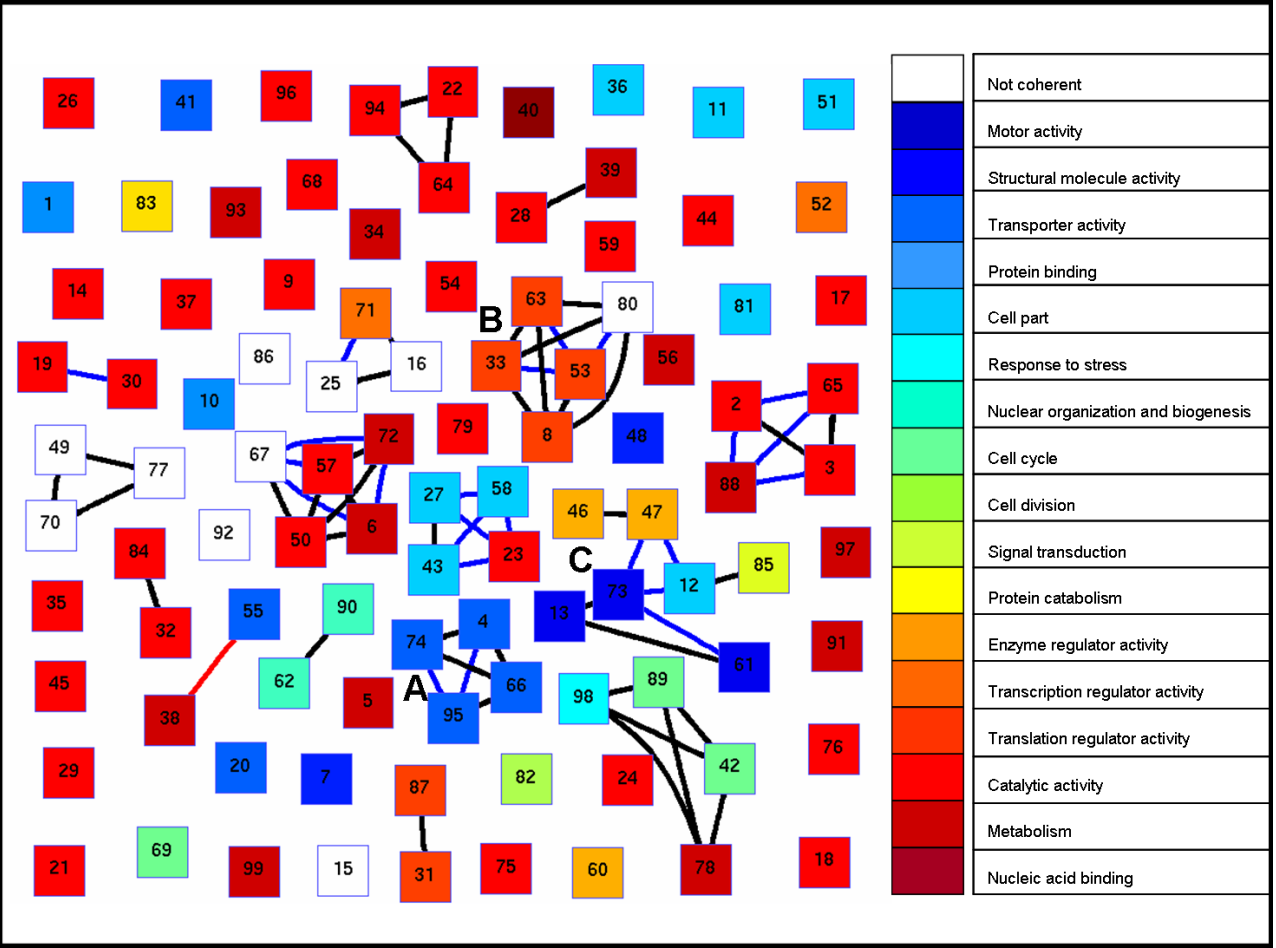
CDS network. Nodes correspond to CDSs (bicliques). Two CDSs are connected by a blue or a red edge if they share at least one domain or protein, respectively; and by a black edge if they share both. CDSs are color coded according to their GO functional enrichment; they are numbered according to their scores, in ascending order. Highlighted are clusters involved in transport **(A)**, translation regulation **(B) **and motor activity, enzyme regulation and cell part **(C).**

In total, we identified 99 significant bicliques in the yeast protein-domain network, each corresponding to a distinct CDS [See Additional file 1]. An overview of the identified bicliques is given in Figure 2. The distributions of biclique sizes in terms of their numbers of proteins and domains are shown in Suppl. Figure S1.

A direct comparison with the combinations in Vogel et al [4] is not possible, as the latter focused on kingdom- rather than organism-specific combinations and examined only contiguous domain combinations. However, our organism-specific application yielded 89 new combinations that were not included in [4]. In particular, 14% of the combinations we identified included more than 3 domains, and 20% of the combinations had at least one non-adjacent occurrence (i.e., a protein in which the combination does not occur contiguously). This demonstrates the utility of our method that can search for CDSs, involving any number of possibly non-adjacent domains.

Some of the CDSs we identified were well supported by previous studies. For example, we identified a combination consisting of the VHS domain (IPR002014) and the UIM (ubiquitin interacting motif) domain (IPR003903). The VHS domain has a membrane targeting role in vesicular trafficking in eukaryotic cells [20]. The UIM domain serves as a ubiquitin binding site [21]. The role of the combination of these domains was studied in the STAM2 (signal-transducing adaptor molecule) protein in [22]. It was shown that both VHS and UIM are required for ubiquitin binding. Specifically, the deletion of any one of these domains was shown to dramatically reduce the ubiquitin binding, whereas a mutant lacking both domains did not bind ubiquitin at all.

As another example, we identified a combination of the motor region of the myosin head (IPR001609) and the IQ calmodulin-binding region (IPR000048) in the myosin family of proteins. These proteins are responsible for actin-based motility in eukaryotic cells, by using ATP hydrolysis to move on actin filaments [23]. They are characterized by three functional subunits: motor head, neck and tail. The head region, located at the N-terminal of the protein, is followed by the neck region. Both regions are well conserved in evolution (in contrast to the tail region) and are responsible for the actin-based movement. The head is composed of a single motor domain, which contains binding sites for ATP and actin [23]. The attached neck is composed of several repeats of the IQ calmodulin-binding region. This domain forms a rigid structure that serves as a mechanical lever, and the number of such domains in the neck determines the length of the lever arm and, hence, the step size of the myosin motor [24].

### Functional annotation of CDSs and the associated proteins

A statistically significant CDS suggests that its associated proteins are involved in similar biological processes. We examined whether the proteins in each of the CDSs exhibited functional coherency according to the gene ontology (GO) annotation (Methods). We found that 89 out of the 99 CDSs (90%) were significantly functionally coherent (Figure 2). To avoid expected matches between the molecular functions of proteins that contain the same set of domains, we repeated the functional coherency analysis when focusing on the GO biological process annotation. 77 out of the 99 CDSs (78%) were found to be significantly coherent in this latter analysis. Finally, we examined the correspondence between the enriched function of a biclique and the annotated functions of its domain. We found that 65 out of the 89 functionally coherent combinations (73%) contained at least one domain whose annotated function in InterPro matched the enriched function. These results serve as a further validation of the significance of the identified combinations, as well as an indication of the biological function that can be attributed to each CDS.

Bicliques sharing domains or proteins were further found to relate in function, as demonstrated by the biclique network in Figure 2. The network exhibits a modular structure where CDSs group together to form clusters with coherent function. For instance, the cluster shown in Figure 2A contains several combinations of the P-type ATPase family domains. These domains are found in cation transport enzymes like sodium, calcium, copper or plasma membrane proton transporting enzymes [25]. In accordance with this functional role, the cluster's proteins are enriched for the terms transport and ATPase activity. As a second example, the cluster in Figure 2B contains combinations of translation and elongation domains. These combinations are found in proteins that assist in delivering aminoacyl tRNA to the acceptor site of the ribosome during protein synthesis, and are also involved in the translocation of the synthesized protein chain from the A to the P site [26]. In accordance with this functional role, the cluster's proteins are enriched for translation factor activity and nucleic acid binding. As a third example, the cluster in Figure 2C spans several different functions. Notably, some of the bicliques in this cluster share versatile domains like the SH3 domain (47,73 and 12), a well known domain that mediates many diverse processes in the cell [27]. Accordingly, these bicliques are enriched with versatile functions such as motor activity, enzyme regulation and cell part. (See Figure S3 for more details).

**Figure 3:**
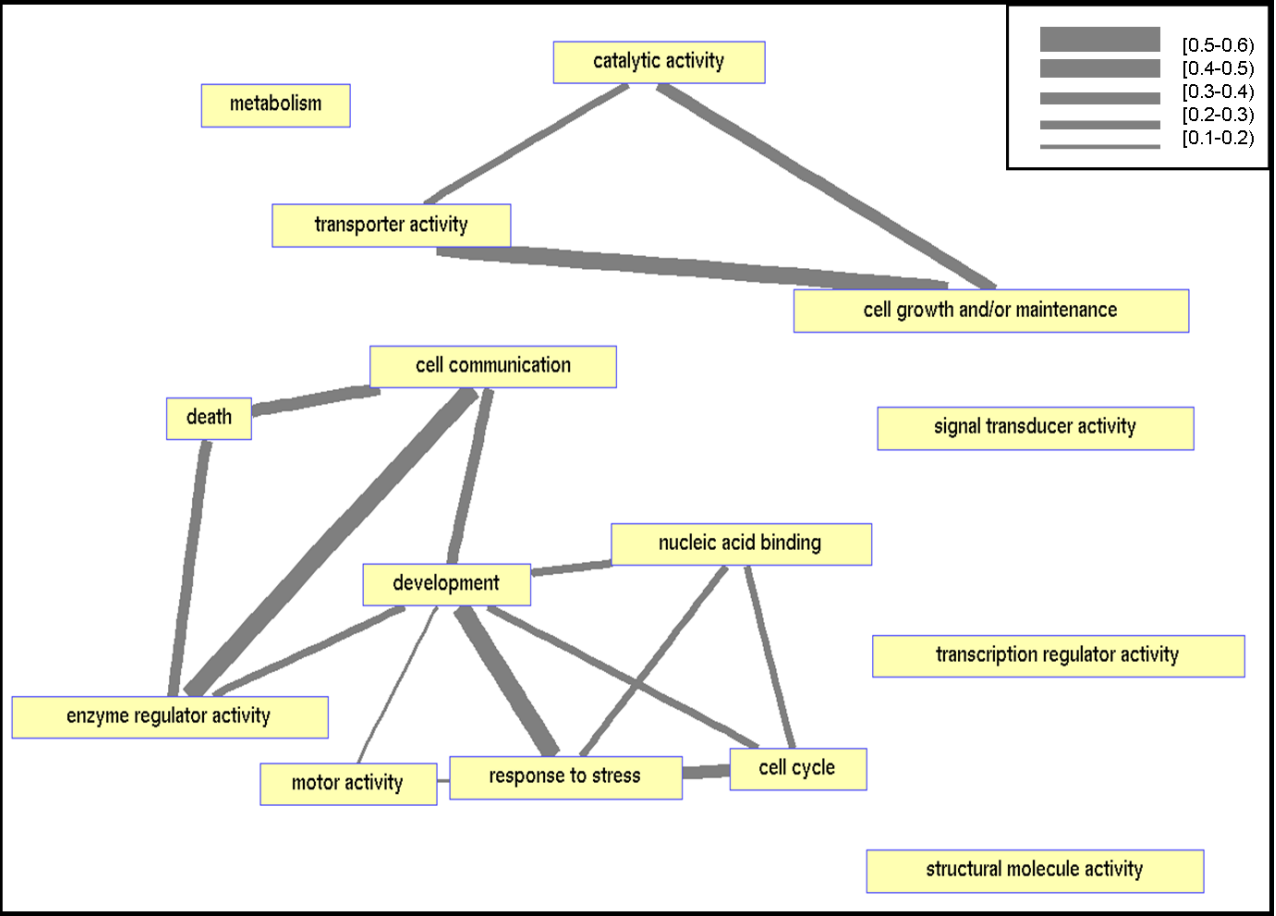
Network of GO functions. Nodes correspond to GO slim terms. Two nodes are connected if the corresponding terms significantly share enriched combinations (hypergeometric *p*-value < 0.05). Edge width is determined by the number of shared proteins divided by the total number of proteins enriched in the corresponding GO terms. Edge widths were binned into 5 categories: [0.1–0.2),[0.2–0.3),[0.3–0.4),[0.4–0.5),[0.5–0.6).

To investigate the relations between the GO terms characterizing the identified CDSs, we also created a network of GO terms, where each node is a GO slim annotation and two nodes are connected if they significantly share enriched combinations (Figure 3 and Methods). In this network one can see a dichotomy of the connected components: one contains basic, essential functions like catalytic or transporter activities, while the other contains higher, sophisticated functions like cell communication, development and cell-cycle. This suggests that each CDS can, in principle, be functionally associated to only one of these classes.

### Protein-protein interactions within bicliques

The functional coherence of proteins within bicliques has led us to investigate the physical connections among them. We expected proteins sharing similar CDSs to interact with one another and to match known yeast complexes. To test whether proteins sharing a particular CDS tend to interact, we compared the fraction of interacting proteins within bicliques to the overall fraction of interacting pairs. We found that proteins sharing a CDS significantly tended to co-interact (*p <*2.7e-11 by a hypergeometric test). As we had a reliability estimate to each reported interaction (Methods), we also compared the reliability distributions of within-biclique interactions and all other interactions. We found that the protein-protein interactions within bicliques were significantly more reliable than other reported interactions (*p < 0.0014 *by a Wilcoxon rank sum test).

As further support for the identified functional relations between proteins sharing a CDS, we tested whether these protein sets are enriched for known protein complexes from the MIPS database [28]. To this end, we computed the fraction of bicliques whose protein sets significantly matched a known complex (Methods). Since the MIPS catalog contains only a limited collection of complexes, we restricted our analysis to bicliques that included at least *t *proteins that were annotated as members of some complex in the MIPS catalog. Overall, 73% (16/22) of the protein sets that had at least two MIPS annotated proteins were significantly enriched for a known complex; and 89% (8/9) of the sets having at least 3 MIPS annotated proteins were enriched.

### Domain age within combinations

**Figure 4:**
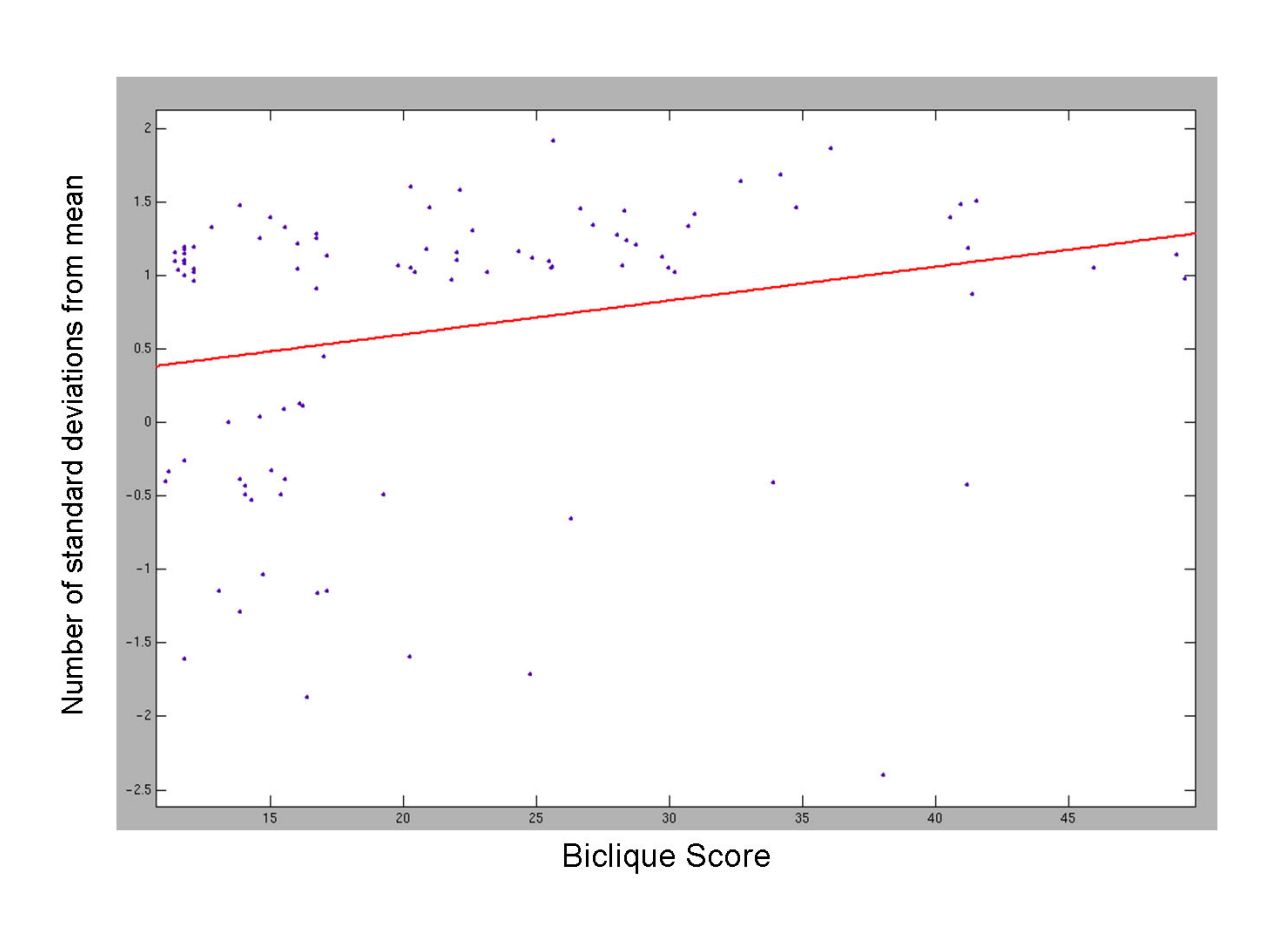
Domain age in combinations. Shown is the fraction of ancient domains in a CDS (number of standard deviations from the mean compared to random domain sets) as a function of the score of the corresponding biclique.

Finally, we studied the age distribution of domains within CDSs. To this end, we classified the yeast domains into *ancient *domains, which are found also in bacteria or archaea, and *new *domains, which are specific to yeast (cf. [35]). CDSs were significantly enriched for ancient domains (*p < 9.3e-7*, see Methods), and there was an evident correlation between the score of a combination (measuring its overrepresentation) and its enrichment level (*p < 0.0047 *by Spearman correlation test), as demonstrated in Figure 4. To provide further support for the observed correlation, we compared the score distributions of bicliques that contain only ancient domains and all other bicliques. We observed a significant difference between the two distributions (*p <*0.0171, by a Wilcoxon rank sum test), as further exemplified in Figure S4.

## Discussion

It has previously been shown that the repertoire of domain combinations in an organism's proteome is restricted to only a small fraction of the set of possible combinations [36]. Here we have used a novel representation of proteins and their domains to investigate the landscape of CDSs. We identified global properties of the protein-domain network, as well as specific highly recurrent and biologically significant CDSs. On the global scale, we have shown that the degree distribution of domains in this network follows a power law, and that highly modular proteins tend to contain abundant domains and proteins with a small amount of domains tend to contain rare domains. On the local scale, we identified highly recurrent CDSs and investigated the sets of proteins and domains that they induce. We observed that the proteins within these sets significantly tended to interact with one another, participate in similar biological processes, and be associated with the same protein complex. The CDSs were shown to include a significantly high fraction of ancient domains that are conserved from bacteria or archaea.

Our analysis relied on the Interpro database, which includes domain annotations from both structure- and sequence-based sources. In order to investigate the influence of the domain type on our results, we devised a rough classification of domains into two categories: A domain is called *sequence-based *if it has a PRINTS [29] or SMART [30] source and *structure-based *if it has a PDB [31], SCOP [32] or CATH [33] source. Out of 1588 domains in our data set, 359 (22.6%) are sequence-based and 975 (61.4%) are structure-based. Some domains have both sequence and structural annotations (19.2%) and some have neither (35.2%). In addition, 1488 (93.7%) of the domains have a PFAM [34] source. As PFAM spans most of the domains in InterPro we focused our analysis on the other two types of domains.

First, we examined whether the functional coherency of bicliques is more prominent for structure- or sequence-based domains. To this end, we defined a biclique as structure-based if all its constituent domains were structure-based, and sequence-based if it contained at least one sequence-based domain. (We used asymmetric definitions here to overcome the 1:3 bias in the numbers of sequence- and structure-based domains in the data, respectively.) We found that 88.5% of the sequence-based bicliques and 92.1% of the structure-based ones are functionally coherent. These rates are comparable to the observed 90% rate when considering all domains. Furthermore, 28% of the functionally coherent bicliques contained at least one sequence-based domain whose annotated function in InterPro matched the biclique's enriched function. Similarly, 70.8% of the functionally coherent bicliques contained at least one structure-based domain whose annotated function matched the enriched function. These percentages nicely match the frequencies of sequence- and structure-based domains in the IntePro domain collection.

Second, we tested the correlation between the domain type and the tendency of a containing protein to interact. Specifically, we compared the fraction of interacting proteins within either sequence- or structure-based bicliques to the overall fraction of interacting pairs. We found that proteins within bicliques of both types significantly tended to co-interact (*p <*0.0059 for sequence-based bicliques and *p <*2.5e-5 for structure-based ones). We conclude that the domain type does not significantly bias the interaction enrichment results.

While our work has produced a valuable list of CDSs in yeast, several of its limitations must be acknowledged. First, our method relies on accurate domain annotation of proteins. Even though InterPro is known to have a low false positive rate (0.2%, see [37]), it is far from complete, covering only 67% (3321/4930) of all SwissProt proteins. Second, in this work we adopted a combinatorial definition of CDSs. That is, a combination was defined as a set of specific domains, and a protein was considered to have this combination only if it contained the exact same set of domains. More general definitions that treat domain occurrences in a probabilistic fashion (e.g., similar to the way that sequence motifs are defined, cf. [38]), together with additional domain data, may uncover additional significant combinations that were missed by the current analysis.

## Methods

### Data acquisition

We downloaded the Interpro [2] domain annotations for SwissProt proteins [39] in the yeast *S. cerevisiae*. We considered only Interpro entries of type domain and family, as described in Cohen-Gihon et al. [35]. In total, our data set contained 1,588 domains and 3,321 yeast proteins.

Protein-protein interaction data were downloaded from the DIP database [40] (July 2005 download), with a total of 15,147 interactions. The interactions were assigned reliability estimates which were computed using a logistic regression model that takes into account the experimental techniques with which each of the interactions was detected [41].

Manually-curated protein complexes were obtained from the MIPS database [28]. We considered all complexes at the leaves of the MIPS hierarchy (excluding category 550 which includes complexes derived by high-throughput experiments). Levels greater than 3 were collapsed to level 3 (i.e., adding their proteins to the corresponding level 3 complex on the path to the root).

### Bipartite graph representation of proteins and domains

We represented the domain content of proteins using a bipartite graph of domain and protein nodes whose edges connect proteins to their constituent domains. We assigned weights to the edges reflecting the chance of observing such edges in a random graph with the same node degrees. Precisely, for an edge connecting nodes of degrees *d' *and *d"*, the edge's weight was set to -log (*d'd"/m*), where *m *represents the total number of edges in the graph [42].

### Analysis of hub proteins in the yeast PPI network

Hub proteins were classified as in Ekman et al. [18] as proteins involved in 8 or more interactions. To compute the enrichment of multi-domain proteins (with at least 3 domains) in hub proteins we used a hypergeometric score. In detail, let *M *denote the total number of proteins, let *K *denote the number of hubs, let *N *denote the number of multi-domain proteins, and let *S *denote the number of proteins that are both multi-domain and hub. Then the corresponding *p*-value is:

p=∑i=Smin⁡(N,K)(Ki)(M−KN−i)(MN)
 MathType@MTEF@5@5@+=feaafiart1ev1aaatCvAUfKttLearuWrP9MDH5MBPbIqV92AaeXatLxBI9gBamXvP5wqSXMqHnxAJn0BKvguHDwzZbqegyvzYrwyUfgarqqtubsr4rNCHbGeaGqiA8vkIkVAFgIELiFeLkFeLk=iY=Hhbbf9v8qqaqFr0xc9pk0xbba9q8WqFfeaY=biLkVcLq=JHqVepeea0=as0db9vqpepesP0xe9Fve9Fve9GapdbaqaaeGacaGaaiaabeqaamqadiabaaGcbaGaemiCaaNaeyypa0ZaaabCaeaadaWcaaqaamaabmaabaqbaeqabiqaaaqaaiabdUealbqaaiabdMgaPbaaaiaawIcacaGLPaaadaqadaqaauaabeqaceaaaeaacqWGnbqtcqGHsislcqWGlbWsaeaacqWGobGtcqGHsislcqWGPbqAaaaacaGLOaGaayzkaaaabaWaaeWaaeaafaqabeGabaaabaGaemyta0eabaGaemOta4eaaaGaayjkaiaawMcaaaaaaSqaaiabdMgaPjabg2da9iabdofatbqaaiGbc2gaTjabcMgaPjabc6gaUjabcIcaOiabd6eaojabcYcaSiabdUealjabcMcaPaqdcqGHris5aaaa@5E54@

### Biclique search

A biclique is defined as a fully connected bipartite subgraph, i.e., a subset of proteins *P *and a subset of domains *D*, such that each protein in *P *contains all the domains in *D*. The score of a biclique reflects the likelihood of observing such a biclique in a random, degree preserving network, and is defined as the sum of the weights assigned to the biclique's edges. We focused on maximal bicliques that contain at least two domains and at least two proteins. To detect the highest-scoring bicliques we adapted the method of Tanay et al. [19,43]. Briefly, for each protein we enumerated all possible combinations of the domains composing it (up to 2^7 ^such combinations are possible in our data). Each such combination was assigned a score according to the weight it induces with respect to the protein (i.e., the sum of weights of the edges connecting the member domains to the protein). Iterating over all the proteins in the data set, for each combination *C *we obtained the total weight of a biclique whose domain set is *C*. By applying the same algorithm to random protein-domain graphs with the same node degrees (see below), we were able to assign an empirical *p*-value to each CDS. The latter was determined by calculating the biclique's ranking in a list of 100 scores, representing the maximum weight obtained in each of 100 random runs. Only bicliques with *p*-value < 0.05 were retained (see Suppl. Figure S2). In total, we identified 99 significant bicliques.

Randomized protein-domain bipartite graphs were created by starting with the original graph, and iteratively shuffling its edges while maintaining node degrees, using the "switch" method [44].

### Functional coherency analysis

Functional coherency of protein sets was based on the Gene Ontology (GO) [45] annotation. The analysis was conducted on the entire GO hierarchy, apart from the analysis related to the GO term network (Figure 3), which was based on 21 representative GO slim terms. For each biclique, we used a hypergeometric score to assess its functional coherency with respect to each of the terms, choosing the highest-scoring one and associating it with the biclique. We assigned each biclique an empirical *p*-value by comparing its hypergeometric score to that of 100 random protein sets of the same size. The resulting *p*-values were further corrected for multiple biclique testing using the false discovery rate (FDR) procedure. Protein sets with corrected *p*-values smaller than 0.05 were considered functionally coherent.

### MIPS complex enrichment analysis

To quantify the correspondence between the bicliques we identified and known complexes from the MIPS database [28], we applied a method described in [46]. Briefly, the set of proteins of each biclique was compared to the known yeast complexes cataloged in MIPS, and the most significant match was selected, using a hypergeometric score. Empirical *p*-values were calculated by comparing the hypergeometric scores to those obtained for random sets of proteins of the same size. These *p*-values were further FDR corrected for multiple testing. The fraction of sets with significant matches (*p < 0.05*) was measured.

### Analysis of domain age distribution

For each set of domains in a biclique, the enrichment of ancient domains was measured using a hypergeometric score and compared to the enrichments under random labelings of domains as ancient and new, respecting the size of each class.

## Authors' contributions

ICG, RS and RN participated in the design of the study and drafted the manuscript. ICG carried out the analyses. All authors read and approved the final manuscript
